# Impact of Blood-Count-Derived Inflammatory Markers in Psoriatic Disease Progression

**DOI:** 10.3390/life14010114

**Published:** 2024-01-12

**Authors:** Oana Mirela Tiucă, Silviu Horia Morariu, Claudia Raluca Mariean, Robert Aurelian Tiucă, Alin Codrut Nicolescu, Ovidiu Simion Cotoi

**Affiliations:** 1 Doctoral School of Medicine and Pharmacy, George Emil Palade University of Medicine, Pharmacy, Science, and Technology of Targu Mures, 540142 Targu Mures, Romania; 2 Dermatology Department, George Emil Palade University of Medicine, Pharmacy, Science, and Technology of Targu Mures, 540142 Targu Mures, Romania; 3 Dermatology Clinic, Mures Clinical County Hospital, 540342 Targu Mures, Romania; 4 Pathophysiology Department, George Emil Palade University of Medicine, Pharmacy, Science, and Technology of Targu Mures, 540142 Targu Mures, Romania; 5 Endocrinology Department, George Emil Palade University of Medicine, Pharmacy, Science, and Technology of Targu Mures, 540142 Targu Mures, Romania; 6 Endocrinology Department, Mures Clinical County Hospital, 540139 Targu Mures, Romania; 7 Agrippa Ionescu Emergency Clinical Hospital, 011773 Bucharest, Romania; 8 Pathology Department, Mures Clinical County Hospital, 540011 Targu Mures, Romania

**Keywords:** psoriasis, inflammation, disease severity, blood markers, inflammatory skin diseases

## Abstract

Psoriasis is a chronic immune-mediated disease, linked to local and systemic inflammation and predisposing patients to a higher risk of associated comorbidities. Cytokine levels are not widely available for disease progression monitoring due to high costs. Validated low-cost and reliable markers are needed for assessing disease progression and outcome. This study aims to assess the reliability of blood-count-derived inflammatory markers as disease predictors and to identify prognostic factors for disease severity. Patients fulfilling the inclusion criteria were enrolled in this study. Patients were divided into three study groups according to disease severity measured by the Body Surface Area (BSA) score: mild, moderate, and severe psoriasis. White blood cell count (WBC), neutrophil-to-lymphocyte ratio (NLR), platelet-to-lymphocyte ratio (PLR), derived neutrophil-to-lymphocyte ratio (d-NLR), systemic immune index (SII), systemic inflammation response index (SIRI), and aggregate index of systemic inflammation (AISI) positively were correlated with disease severity (*p* < 0.005). d-NLR, NLR, and SII are independent prognostic factors for mild and moderate psoriasis (*p* < 0.05). d-NLR is the only independent prognostic factor for all three study groups. Moderate psoriasis is defined by d-NLR values between 1.49 and 2.19. NLR, PLR, d-NLR, MLR, SII, SIRI, and AISI are useful indicators of systemic inflammation and disease severity in psoriasis.

## 1. Introduction

Psoriasis is a chronic immune-mediated disease characterized by abnormal keratinocyte proliferation and the activation of innate and adaptative immune responses, with consequent immune cells infiltrating the skin. Even though it was initially considered a cutaneous disease, the concept of “psoriatic disease” [1] has been established, indicating that it extends beyond the skin level, affecting the joints, blood vessels, and the heart. As such, psoriasis is linked to local and systemic inflammation, predisposing patients to a higher risk of developing various comorbidities, such as arthritis, metabolic syndrome, inflammatory bowel syndrome, or cardiovascular disease [2]. 

The increased inflammatory state in psoriasis is partly due to cytokines and adipokines produced by the visceral adipose tissue [3], explaining, therefore, the increased risk of obesity these patients have, and partly due to Th-17 and consequent cytokines overexpression in the skin and joints of psoriasis patients [4]. An enhanced oxidative stress is linked to psoriasis, with increased glutathione-S transferase activity and decreased superoxide dismutase and glutathione peroxidase activity [5] in the psoriatic epidermis. Moreover, by magnifying inflammatory processes, psoriasis alters endothelial cell function by raising endothelin-1 and 2 plasmatic levels [6] and increases the risk of atherosclerosis and major cardiovascular events through the psoriatic march [7]. An increased and prolonged systemic inflammatory status in psoriasis may therefore be an important determinant in disease severity, progression, and outcome. 

Clinically defined by erythema, scaling, and induration, psoriasis diagnosis is mainly based on clinical examinations and secondarily on anamnesis and histopathological criteria. Various tools have been developed for clinically grading psoriasis severity, such as the Psoriasis Area and Severity Index (PASI), the Body Surface Area score (BSA), or combined scores such as Physician’s Global Assessment (PGA) × BSA, modified PASI (mPASI), or psoriasis log-based area (PLASI). Nevertheless, each of these scales provides an accurate and reproducible psoriasis severity assessment [8,9].

Moreover, despite the rapidly growing knowledge regarding cytokines and interleukins’ involvement in psoriasis, they prove to be expensive and scarcely available to routinely assess inflammatory state in psoriasis. A clear-cut and prompt analysis of interleukin levels might be offered in the future by validated novel point-of-care devices using label-free electrochemical immunosensors, such as one determining IL-6 levels [10]. As such, a proper identification and comprehensive analysis of simple, low-cost, and widely available markers of systemic inflammation may provide much-needed additional data for a proper diagnosis, assessment, and treatment of patients with psoriasis. 

Various blood-count-derived inflammatory markers have gained interest in recent years due to their high availability. The most extensively studied, the neutrophil-to-lymphocyte ratio (NLR) and platelet-to-lymphocyte ratio (PLR), were described with skin diseases such as hidradenitis suppurativa [11,12], atopic dermatitis [13], or bullous pemphigoid [14], but also related to various cancers [15,16], chronic obstructive pulmonary disease [17], and diabetes [18]. The aggregate index of systemic inflammation (AISI), a potential biomarker of pulmonary fibrosis [19] and hypertension-induced cardiovascular mortality [20], has never been assessed until now in relationship with psoriasis severity. The derived neutrophil-to-lymphocyte ratio (d-NLR) has proved its usefulness merely by referring to cancer prognosis [21,22,23]. The platelet-to-monocyte ratio (PMR) is a novel prognostic factor in liver cirrhosis [24], dyslipidemia [25], and T-cell lymphoma [26].

This study aims to assess the usefulness of blood-count-derived inflammatory markers as indicators of disease severity in psoriasis. Moreover, as far as we know, no study has assessed until now the reliability of AISI, d-NLR, and PMR as predictors of psoriasis severity.

## 2. Materials and Methods

### 2.1. Study Population 

We conducted an observational retrospective study including patients diagnosed with psoriasis vulgaris at Mures Clinical County Hospital’s Dermatology Department between January 2017 and December 2022. Patients over the age of 18, diagnosed with psoriasis vulgaris during the aforementioned period, and with available data regarding disease severity and laboratory investigations were included in this study. The following were considered exclusion criteria: patients of pediatric age and diagnosed with other types of psoriasis, with no information regarding disease severity and laboratory investigation available; and patients with diabetes, active infections, malignant tumors, cardiovascular or liver disease at time of enrollment, and those who, three months before enrollment, had undergone systemic treatment with one of the following drugs: steroids, antimetabolites (methotrexate and azathioprine), calcineurin inhibitors (cyclosporine), or innovative drugs (any type of biologics and phosphodiesterase-4 inhibitors).

### 2.2. Data Collection

Patients’ demographic, clinical, and laboratory data were gathered using the hospital’s electronic databases. The Body Surface Area (BSA) score was used to assess psoriasis severity and interpreted in the following manner: mild (BSA < 5%), moderate (5% ≤ BSA < 10%), and severe (BSA > 10%). The laboratory parameters assessed were the following: complete white blood cell count (WBC), leucocyte subsets (neutrophils, lymphocytes, and monocytes) count, platelet count, and erythrocyte sedimentation rate (ESR). For patients who presented more than once to our clinic during the study period, data were collected based on the first presentation.

### 2.3. Biomarkers

Whole blood venous samples were collected in the morning, after an overnight fast, and analyzed with a Mindray BC-6200 automatic hematology analyzer (Mindray Medical International Limited, Shenzhen, China). The following blood-count-derived inflammatory markers were evaluated: neutrophil-to-lymphocyte ratio (NLR), derived neutrophil-to-lymphocyte ratio (d-NLR), monocyte-to-lymphocyte ratio (MLR), platelet-to-monocyte ratio (PMR), systemic immune index (SII), systemic inflammation response index (SIRI), and aggregate index of systemic inflammation (AISI). Table 1 depicts the formulas for the parameters mentioned above.

### 2.4. Study Outcome

In this paper, we sought to primarily determine whether blood-count-derived inflammatory markers may be used as prognostic factors of disease severity in psoriasis. Based on severity, patients were divided into three study groups: mild (BSA < 5%), moderate (5% ≤ BSA < 10%), and severe psoriasis (BSA > 10%). Second, we aimed to identify independent prognostic factors for disease severity. As far as we are aware, this is the first paper to analyze the overall relationship between d-NLR, PMR, AISI, and psoriasis severity.

### 2.5. Statistical Analysis

The MedCalc Statistic software for Windows, version 22.014, was used for the statistical analysis. A Shapiro–Wilk test was used to evaluate data normality. Data are presented as absolute counts and proportions for categorical variables, and as median or mean with standard deviations (SD) for continuous variables. The difference between study groups was assessed by the Mann–Whitney test for continuous variables, and by the Chi-square test for categorical ones. Correlations were assessed by Spearman’s correlation coefficient. One-way ANOVA was used to compare data in the three study groups. Post hoc Bonferroni analysis was used to assess multiple comparisons between groups. The performance of inflammatory markers in predicting disease severity was assessed using receiver operating characteristics (ROC) curve analysis and the area under the ROC curves (AUCs). The optimal cut-off values for relevant systemic inflammatory markers were determined using the Youden Index. Different parameters were compared using the DeLong Z test. Multivariate logistic regression was conducted to identify independent prognostic factors associated with psoriasis severity. The goodness of fit for the regression model was assessed using the Hosmer–Lemeshow test. *p* < 0.05 was considered statistically significant throughout all the analyses.

## 3. Results

### 3.1. Patients’ Clinical Profile

A total of 366 patients were included in this study. The majority were males (n = 219) and had a mean age of 54.48 ± 16.48. In terms of disease severity, 180 had mild disease, 143 had moderate psoriasis, and 43 presented with a severe course of disease. No statistically significant differences were identified between the three study groups in terms of age, gender, platelet, lymphocyte, and neutrophil count, nor for PMR and ESR values. On the other hand, WBC, neutrophil count, NLR, d-NLR, SII, SIRI, and AISI (*p* ≤ 0.001), PLR (*p* = 0.002), and MLR (*p* = 0.04), respectively, were significantly different between the three study groups (as seen in Table 2).

Subsequently, post hoc Bonferroni analysis (Table 3) revealed that patients with moderate psoriasis had significantly higher values of WBC (*p* = 0.004), neutrophil count (*p* = 0.003), NLR (*p* = 0.01), d-NLR (*p* = 0.02), and SII (*p* = 0.009) compared to those with mild disease and significantly lower neutrophil count (*p* = 0.01) and d-NLR (*p* = 0.007) values than those with severe disease. 

### 3.2. Serological Markers and Disease Severity

Further analysis was conducted on the association between serological markers and disease severity. As the disease advanced, platelet and neutrophil count, PLR, NLR, d-NLR, PMR, SII, SIRI, and AISI increased. A constant gradual decrease was noted in the lymphocyte count. As depicted in Table 4, WBC, neutrophil count, PLR, NLR, d-NLR, MLR, SII, SIRI, and AISI significantly and positively correlated with disease severity. NLR exhibited the strongest correlation (r = 0.30). On the other hand, no correlation was identified for PMR, platelet, lymphocyte, and monocyte count.

### 3.3. Performance of Blood-Count-Derived Inflammatory Markers in Evaluating Disease Severity

The diagnostic performance of different markers is shown in Table 5.

**Figure 1:**
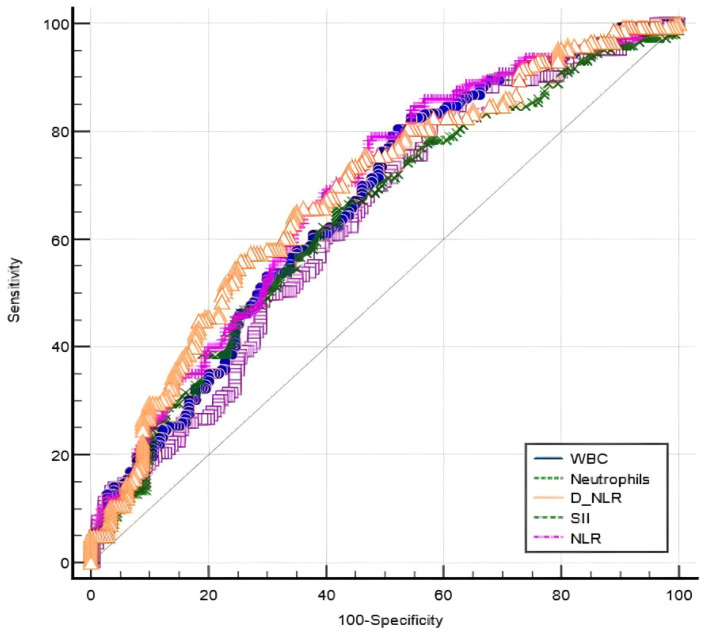
ROC comparison of WBC, neutrophils, NLR, d-NLR, and SII in predicting moderate psoriasis.

For discriminating between moderate and mild psoriasis, the AUC of WBC was 0.637 with a cut-off value of 6.25, while the neutrophil count AUC was 0.662 with a cut-off value of 3.64. On the same matter, the AUC of NLR was 0.687 with a cut-off value of 2.35, while d-NLR and SII predicted moderate psoriasis at, respectively, an AUC of 0.640 and a cut-off value of 65.73, and an AUC of 0.683 and a threshold value of 408.8. As for sensitivity, WBC was the highest, while specificity was the highest for NLR. Comparing AUCs of various markers for predicting a moderate course of disease, the AUC of NLR was the highest, but similar to those of WBC (*p* = 0.11), neutrophil count (*p* = 0.28), and SII (*p* = 0.81) and significantly different from that of d-NLR (*p* < 0.001) (Figure 1).

If referring to differentiating severe psoriasis from moderate disease, d-NLR significantly (*p* = 0.03) predicted severe psoriasis at an AUC of 0.598 and a cut-off value of 2.18. Nevertheless, regarding neutrophil count, due to the non-significant level (*p* = 0.55), the obtained AUC of 0.527 and the cut-off value of 5.66 cannot be considered reliable markers in discriminating between severe and moderate psoriasis. 

### 3.4. The Dependability of Blood-Count-Derived Inflammatory Markers for Predicting Disease Severity

In a multivariate logistic regression model, patients with d-NLR (OR: 0.16, *p* < 0.001), NLR (OR: 4.13, *p* < 0.001), and SII (OR: 1, *p* = 0.046) above the cut-off values of 1.49, 2.35, and 408.8, respectively, have a higher risk of presenting with moderate psoriasis. Moreover, values for the aforementioned parameters below the cut-off value are significant predictors of mild psoriasis, as seen in Table 6. If referring to severe psoriasis, a higher level of d-NLR (OR: 0.69, *p* = 0.049) is an independent predictor of severe psoriasis. 

## 4. Discussion

Early detection and an overall assessment of systemic inflammation are of great importance when referring to psoriasis. Psoriatic patients should benefit from an integrated, multidisciplinary-based therapeutical approach, taking into account the elevated risk of associated comorbidities this disease imposes.

Previous studies found that laboratory markers like NLR, PLR, MLR, and SII are associated with psoriasis or its severity, as well as with malignant tumors or other autoimmune diseases [27,28]. Currently, even though NLR and PLR are the most extensively studied, no consistent marker for assessing disease severity was reported, probably due to inconsistent study groups and racial variations, highlighting the need for more extensive studies on the subjects. NLR was reported to be higher in patients with psoriasis compared to controls [29] and a strong predictor of psoriatic arthritis [30]. Additionally, these markers can be used as indicators of all-cause mortality [31] and cardiovascular disease [32]. We have recently shown their reliability in assessing liver fibrosis [33] in psoriasis. NLR and PLR decreased in a similar form to the C-reactive protein (CRP) in Japanese patients treated with all kinds of biologics [34], and can, therefore, be useful in assessing the response to systemic treatment. Nevertheless, larger studies focusing on response to various kinds of biologics are needed in the future. 

The present study identified that platelets and leucocytes subsets are potential contributors to psoriatic systemic inflammation. As depicted in Table 2 and Table 4, psoriatic severity is significantly positively correlated with WBC, neutrophil count, PLR, NLR, d-NLR, MLR, SII, SIRI, and AISI. Endothelial cells, epidermal keratinocytes, and dermal dendritic cells are involved in producing high levels of TNF-α, while Th17 cells, neutrophils, mast cells, and macrophages produce interleukin-17. Additionally, neutrophils promote increased production of reactive oxygen species [35] and are a major source of antimicrobial proteins [36] and lipocalin 2 [37], further sustaining systemic inflammation. Patients with psoriasis exhibit higher levels of advanced oxidation protein products [38,39] and catalase compared to controls [39], with additional differences between genders [40] supporting once more the need for a tailored therapeutical approach to each patient. Epidermal infiltration of neutrophils, a histopathological marker of psoriasis [41], with consequent activation, leads to the formation of neutrophil extracellular traps (NETs), which further interact directly with keratinocytes and heighten the IL-17A response [42]. In a study published by Rocha-Pereira et al. [43], psoriasis was characterized by an increased neutrophil count, with significantly higher levels in active psoriasis compared to inactive disease [43]. In our study, a constant gradual decrease was noted in the lymphocyte count as the severity increased. This paradoxical reaction is most likely the reverse effect of an increased influx of lymphocytes into the skin in patients with severe disease. 

Moreover, as the disease progressed, platelet count increased in our study groups. Even though originally platelets were considered to interfere mainly with hemostasis, additional data highlight their involvement in inflammation progression and immunological processes [44]. Elevated platelet count may be due to bone marrow hyperproduction consecutive to their accumulation at inflammation sites, but also because TNF-α can directly activate platelets. Monocyte count was higher in severe forms compared to milder ones. They are responsible for producing interleukin-1 (IL-1) and interleukin-6 (IL-6), TNF-α [45], while immune cells derived from monocytes are key factors in interleukin-23 (IL-23)-driven psoriatic inflammation [46]. Nevertheless, it is worth mentioning that, when referring to our study and monocyte-derived markers, MLR varied significantly between the three study groups (*p* = 0.04), but PMR did not (*p* = 0.75). PMR values were the highest in patients presenting with mild disease, suggesting that this marker might not be reliable in assessing inflammatory status in psoriasis. 

Thus, NLR, PLR, d-NLR, MLR SII, SIRI, and AISI seem to adequately reflect this involvement and, therefore, systemic inflammation in psoriasis. NLR exhibited the strongest correlation with disease severity (r = 0.30), followed by SII (r = 0.29), SIRI, and AISI (r = 0.28). PLR and MLR individually had a weak association with disease severity (r = 0.14 and r = 0.13, respectively). As such, composite markers, like SII, SIRI, and AISI, which incorporate various cell subsets (neutrophils, lymphocytes, monocytes, and/or platelets), may become more promising prognostic factors. 

Patients with moderate psoriasis had significantly higher WBC, neutrophils count, NLR, d-NLR, and SII values compared to milder forms and lower neutrophils count and d-NLR values if compared to those with severe forms. In differentiating between moderate and milder forms of psoriasis, all the aforementioned parameters proved to be reliable and statistically significant taking into account the AUC (*p* < 0.001). Based on the AUC values, NLR is the most reliable factor in discriminating between moderate and mild psoriasis (AUC = 0.687) with a cut-off value of 2.35. Moderate psoriasis is defined by WBC above 6.25, neutrophil count above 3.64, NLR values above 2.35, d-NLR above 1.49, and SII above 408.8. Moreover, after running a multivariate regression analysis model, d-NLR (OR: 0.16), NLR (OR: 4.13), and SII proved to be significant (*p* < 0.001, *p* < 0.001, and *p* = 0.043, respectively) of a moderate course of disease. NLR is proven to be the strongest predictor of moderate psoriasis.

If talking about mild psoriasis, it should be defined by WBC below 6.25, neutrophil count below 3.64, NLR below 2.35, d-NLR below 1.49, and SII below 408.8. Regarding prognostic factors, our study identified that d-NLR (OR: 6.15), NLR (OR: 4.13), and SII (OR: 0.99) are significant indicators (*p* < 0.001, *p* < 0.001, and *p* = 0.046, respectively) of mild psoriasis.

On the other hand, when referring to severe psoriasis, our analysis identified that it can be differentiated from moderate psoriasis based on an AUC of 0.598 with a consequent calculated cut-off value of 2.18. With a *p*-value of 0.55, the neutrophil count cannot be considered a reliable marker in discriminating between severe and moderate psoriasis. Therefore, taking all of this into account, our study suggests that, referring to moderate psoriasis, it should be defined by d-NLR values between 1.49 and 2.18, with values below the lower limit of the interval indicating mild psoriasis, and higher values indicating severe psoriasis. 

Out of all parameters overall identified as prognostic factors of disease severity, d-NLR is the only one linked to all three study groups. To the best of our knowledge, the reliability of d-NLR as an inflammatory marker in psoriasis has not been assessed until now. Unlike NLR, d-NLR also incorporates monocytes and other granulocytes, because it is calculated by dividing the neutrophil count by the difference between WBC and the neutrophil count (see Table 1). In an increased systemic inflammatory state, as in psoriasis, immature neutrophils can be released, leading to a rapid increase in neutrophil count. As such, compared to NLR, d-NLR reflects more accurately this negative inflammation, limits potential bias, and, in our study, proves to be the most reliable blood-count-derived inflammatory marker to predict disease severity.

Although our study showed a definite association between blood-count-derived inflammatory markers and disease severity in psoriasis, there are some limitations worth mentioning. First of all, it was a single-center retrospective study; this should be addressed in future studies by a prospective enrollment of the patients who should originate from multiple dermatologic centers, as such diminishing also the influence of geographical factors. Second, disease onset was not addressed; therefore, future work should additionally focus on this matter, as well as on patients’ behavioral patterns. Lastly, disease severity was assessed using only one scale, the BSA score. Our study was based on the BSA score and not the PASI score, because the latter one, even though frequently used, takes into account various parameters such as induration, erythema, and scaling, all dependent up to a certain point on the experience of the examiner, and these could not be controlled in this study, all the more so due to the retrospective manner of enrollment. Future ideas in this matter might refer to a comprehensive disease severity assessment using composite scores.

## 5. Conclusions

NLR, PLR, d-NLR, MLR SII, SIRI, and AISI are useful indicators of systemic inflammation and disease severity in psoriasis. Additionally, d-NLR accurately reflects the negative inflammation associated with this disease and proves to be the most reliable blood-count-derived inflammatory marker in predicting disease severity across all three study groups. 

Referring to easily obtainable and low-cost markers, the results of our study comprehensively highlight the link between systemic inflammation and psoriasis progressions. Reporting for the first time the usefulness of d-NLR and AISI in appreciating and predicting disease severity, our results emphasize the usefulness of blood-count-derived inflammatory markers in a proper patient assessment monitoring disease course.

## Figures and Tables

**Table 1:** Formulas of analyzed biomarkers.

Marker	Formula
NLR	Neutrophil count/lymphocyte count [×10^3^/μL]
MLR	Monocyte count/lymphocyte count [×10^3^/μL]
d-NLR	Neutrophil count/(WBC − neutrophil count) [×10^3^/μL]
PLR	Platelet count/lymphocyte count [×10^3^/μL]
PMR	Platelet count/monocyte count [×10^3^/μL]
SII	(Neutrophil count × platelet count)/lymphocyte count [×10^3^/μL]
SIRI	(Neutrophil count × monocyte count)/lymphocyte count [×10^3^/μL]
AISI	(Neutrophil count × monocyte count × platelet count)/lymphocyte count [×10^3^/μL]

NLR, neutrophil-to-lymphocyte ratio; d-NLR, derived neutrophil-to-lymphocyte ratio; PLR, platelet-to-lymphocyte ratio; MLR, monocyte-to-lymphocyte ratio; SII, systemic immune inflammation index; SIRI, systemic inflammation response index; AISI, aggregate index of systemic inflammation.

**Table 2:** The characteristics of the study population.

Variables *	All Patients	Mild Disease (n = 180)	Moderate Disease (n = 143)	Severe Disease (n = 43)	*p*-Value
Age	54.48 ± 16.48	53.86 ± 17.46	57.51 ± 12.89	54.35 ± 16.18	0.43
Gender					
Male	219	101 (56%)	91 (64%)	27 (63%)	0.36
Female	147	79 (44%)	52 (36%)	16 (37%)	
WBC	7.49 [7.15–7.83]	6.77 [6.38–7.26]	8.08 [7.60–8.73]	7.98 [7.50–8.42]	<0.001
Platelets	238.25 [230.93–243.69]	231.70 [220-.95–240.22]	243.20 [221.87–265.52]	245 [236.51–259.56]	0.11
Neutrophils	4.42 [4.22–4.69]	3.80 [3.44–4.25]	4.68 [4.39–5.26]	4.96 [4.57–5.45]	<0.001
Lymphocytes	2.10 [1.97–2.23]	2.22 [1.99–2.30]	2.16 [1.91–2.34]	1.97 [1.79–2.20]	0.12
Monocytes	0.51 [0.48–0.53]	0.49 [0.45–0.52]	0.52 [0.47–0.56]	0.53 [0.48–0.57]	0.59
PLR	114.96 [110.45–121.10]	108.07 [100.89–115.10]	110.75 [96.66–126.26]	129.79 [117.02–135.15]	0.002
NLR	2.05 [1.90–2.19]	1.73 [1.59–1.88]	2.23 [1.93–2.66]	2.53 [2.20–2.79]	<0.001
d-NLR	1.55 [1.44–1.65]	1.37 [1.29–1.49]	1.45 [1.36–1.82]	1.80 [1.62–1.95]	0.001
MLR	0.23 [0.22–0.25]	0.22 [0.21–0.25]	0.25 [0.23–0.28]	0.24 [0.22–0.26]	0.04
PMR	475.02 [450.39–508.40]	479.08 [440.36–526.90]	467.59 [400–539.75]	476.42 [437.62–524.45]	0.75
SII	478.52 [453.65–521.85]	404.61 [357.44–446.03]	541.21 [464.89–602.06]	560.46 [518.95–646.30]	<0.001
SIRI	1.03 [0.93–1.09]	0.86 [0.79–0.92]	1.23 [1.01–1.44]	1.27 [1.07–1.37]	0.001
AISI	255.83 [229–273.18]	196.96 [168.52–221.22]	287.76 [258.18–331.08]	293.10 [272.01–335.63]	0.001
ESR	15 [12.8–17]	12.80 [11.80–15.80]	16.20 [12.20–20.98]	18 [14–20]	0.17

WBC, white blood cell count; PLR, platelet-to-lymphocyte ratio; NLR, neutrophil-to-lymphocyte ratio; d-NLR, derived neutrophil-to-lymphocyte ratio; MLR, monocyte-to-lymphocyte ratio; PMR, platelet-to-monocyte ratio; SII, systemic immune inflammation index; SIRI, systemic inflammation response index; AISI, aggregate index of systemic inflammation; ESR, erythrocyte sedimentation rate; * refer to Table 1 for measurement units.

**Table 3:** Pairwise comparison of blood-count-derived inflammatory markers.

Parameter *	Mild Psoriasis		Moderate Psoriasis		Severe Psoriasis	
	vs. Moderate Psoriasis	vs. Severe Psoriasis	vs. Mild Psoriasis	vs. Severe Psoriasis	vs. Mild Psoriasis	vs. Moderate Psoriasis
WBC	0.004	0.32	0.004	0.06	0.32	0.06
Neutrophils	0.003	1	0.003	0.01	1	0.01
PLR	0.07	0.32	0.07	1	0.32	1
NLR	0.01	0.96	0.01	0.11	0.86	0.11
d-NLR	0.02	1	0.02	0.007	1	0.007
MLR	1	1	1	1	1	1
SII	0.009	0.34	0.009	0.12	0.34	0.12
SIRI	0.18	1	0.18	0.26	1	0.26
AISI	0.06	1	0.06	0.37	1	0.37

WBC, white blood cell count; PLR, platelet-to-lymphocyte ratio; NLR, neutrophil-to-lymphocyte ratio; d-NLR, derived neutrophil-to-lymphocyte ratio; MLR, monocyte-to-lymphocyte ratio; SII, systemic immune inflammation index; SIRI, systemic inflammation response index; AISI, aggregate index of systemic inflammation; * refer to Table 1 for measurement units.

**Table 4:** Correlation between disease severity and the analyzed markers.

Marker *	r	*p*-Value	Marker	r	v-Value
WBC	0.25	<0.001	MLR	0.13	0.01
Neutrophil count	0.26	<0.001	SII	0.29	<0.001
PLR	0.14	0.005	SIRI	0.28	<0.001
NLR	0.30	<0.001	AISI	0.28	<0.001
d-NLR	0.18	<0.001	ESR	0.15	<0.001

WBC, white blood cell count; PLR, platelet-to-lymphocyte ratio; NLR, neutrophil-to-lymphocyte ratio; d-NLR, derived neutrophil-to-lymphocyte ratio; MLR, monocyte-to-lymphocyte ratio; SII, systemic immune inflammation index; SIRI, systemic inflammation response index; AISI, aggregate index of systemic inflammation; ESR, erythrocyte sedimentation rate; * refer to Table 1 for measurement units.

**Table 5:** Predictive performance of blood-count-derived markers.

Moderate vs. Mild Disease							
Parameter *	AUC (95% CI)	*p*-Value	Cut-Off	Se (%)	Sp (%)	Youden Index J	*p*-Value *
WBC	0.637 [0.582–0.689]	<0.001	6.25	82.52	42.22	0.25	0.11
Neutrophil count	0.662 [0.607–0.713]	<0.001	3.64	80.42	47.78	0.28	0.28
NLR	0.687 [0.633–0.737]	<0.001	2.35	55.94	74.44	0.30	-
d-NLR	0.640 [0.585–0.692]	<0.001	1.49	65.73	58.33	0.24	<0.001
SII	0.683 [0.629–0.733]	<0.001	408.8	79.02	52.22	0.31	0.81
**Severe vs. moderate disease**							
Parameter *	**AUC (95% CI)**	***p*** **-value**	**Cut-Off**	**Se (%)**	**Sp (%)**	**Youden Index J**	***p*-value**
Neutrophil count	0.527 [0.453–0.601]	0.55	5.66	76.74	38.46	0.15	-
d-NLR	0.598 [0.524–0.669]	0.03	2.18	90.70	31.47	0.22	-

Se: sensitivity; Sp: specificity; * Compared to NLR; WBC, white blood cell count; NLR, neutrophil-to-lymphocyte ratio; d-NLR, derived neutrophil-to-lymphocyte ratio; MLR, monocyte-to-lymphocyte ratio; SII, systemic immune inflammation index; * refer to Table 1 for measurement units.

**Table 6:** Predictors of disease severity in psoriasis.

Parameter *	OR	95% CI	*p*-Value
**Mild psoriasis**			
d-NLR	6.15	2.53–14.91	<0.001
NLR	0.24	0.12–0.48	<0.001
SII	0.99	0.99–1.02	0.043
**Moderate psoriasis**			
d-NLR	0.16	0.07–0.39	<0.001
NLR	4.13	2.11–8.11	<0.001
SII	1	1–1.03	0.046
**Severe psoriasis**			
d-NLR	0.69	0.47–1.01	0.049

d-NLR, derived neutrophil-to-lymphocyte ratio; NLR, neutrophil-to-lymphocyte ratio; SII, systemic immune inflammation index; * refer to Table 1 for measurement units.

## Data Availability

All data presented can be made available upon request.
